# Microenvironment Remodeling and Subsequent Clinical Implications in Diffuse Large B-Cell Histologic Variant of Richter Syndrome

**DOI:** 10.3389/fimmu.2020.594841

**Published:** 2020-12-14

**Authors:** Hélène Augé, Anne-Béatrice Notarantonio, Romain Morizot, Anne Quinquenel, Luc-Matthieu Fornecker, Sébastien Hergalant, Pierre Feugier, Julien Broséus

**Affiliations:** ^1^ Inserm UMRS1256 Nutrition-Génétique et Exposition aux Risque Environnementaux (N-GERE), Université de Lorraine, Nancy, France; ^2^ Université de Lorraine, CHRU-Nancy, service d’hématologie clinique, pôle spécialités médicales, Nancy, France; ^3^ UMR7365 Ingénierie Moléculaire et Physiopathologie Articulaire (IMOPA), CNRS, Université de Lorraine, Nancy, France; ^4^ Département d'hématologie, Université de Reims Champagne-Ardenne, Reims, France; ^5^ Département d'hématologie clinique, Centre Hospitalier Universitaire de Reims, Reims, France; ^6^ Université de Strasbourg, Inserm, IRFAC/UMR-S1113, Strasbourg, France; ^7^ Département d'hématologie clinique, Institut de Cancérologie Strasbourg Europe, Strasbourg, France; ^8^ Université de Lorraine, CHRU-Nancy, service d’hématologie biologique, pôle laboratoires, Nancy, France

**Keywords:** Richter syndrome, chronic lymphocytic leukemia, diffuse large B-cell lymphoma, genomics, microenvironment, immune checkpoint, immune checkpoint inhibitor

## Abstract

**Introduction:**

Richter Syndrome (RS) is defined as the development of an aggressive lymphoma in the context of Chronic Lymphocytic Leukemia (CLL), with a Diffuse Large B-Cell Lymphoma (DLBCL) histology in 95% cases. RS genomic landscape shares only a few features with *de novo* DLBCLs and is marked by a wide spectrum of cytogenetic abnormalities. Little is known about RS microenvironment. Therapeutic options and efficacy are limited, leading to a 12 months median overall survival. The new targeted treatments usually effective in CLL fail to obtain long-term remissions in RS.

**Methods:**

We reviewed available PubMed literature about RS genomics, PD-1/PD-L1 (Programmed Death 1/Programmed Death Ligand 1) pathway triggering and subsequent new therapeutic options.

**Results:**

Data from about 207 patients from four landmark papers were compiled to build an overview of RS genomic lesions and point mutations. A number of these abnormalities may be involved in tumor microenvironment reshaping. T lymphocyte exhaustion through PD-L1 overexpression by tumor cells and subsequent PD-1/PD-L1 pathway triggering is frequently reported in solid cancers. This immune checkpoint inhibitor is also described in B lymphoid malignancies, particularly CLL: PD-1 expression is reported in a subset of prolymphocytes from the CLL lymph node proliferation centers. However, there is only few data about PD-1/PD-L1 pathway in RS. In RS, PD-1 expression is a hallmark of recently described « Regulatory B-cells », which interact with tumor microenvironment by producing inhibiting cytokines such as TGF-β and IL-10, impairing T lymphocytes anti-tumoral function. Based upon the discovery of high PD-1 expression on tumoral B lymphocyte from RS, immune checkpoint blockade therapies such as anti-PD-1 antibodies have been tested on small RS cohorts and provided heterogeneous but encouraging results.

**Conclusion:**

RS genetic landscape and immune evasion mechanisms are being progressively unraveled. New protocols using targeted treatments such as checkpoint inhibitors as single agents or in combination with immunochemotherapy are currently being evaluated.

## Introduction

Chronic Lymphocytic Leukemia (CLL) is the most frequent leukemia in Western countries (1). Although considered to be an indolent B-cell neoplasm, CLL actually represents a wide spectrum of diseases from a clinical, biological and prognostic point of view, ranging from non-progressive or poorly progressive to aggressive courses (2). CLL prognosis was first assessed using clinical classifications (3, 4) to which cytogenetic and molecular data were later added. CLLs are distributed among 2 major molecular subtypes that differ in their degree of somatic hypermutations in the *IGHV* (*Immunoglobulin Heavy chain Variable domain*) gene. The *IGHV* unmutated CLLs (U-CLLs), share more than 98% homology with germline sequence and are associated with a worse prognosis than the *IGHV* mutated CLLs (M-CLLs) (5, 6). The combinatorial diversity of VDJ segments at the origin of rearrangements of the *IGHV* gene continuously generates a vast repertoire of B lymphocytes, all different, characterized by a single B-Cell receptor (BCR). A third of the CLLs have been shown to have a stereotypic BCR, meaning that a significant part of B lymphocytes express a restricted immunoglobulin gene repertoire leading to the expression of highly similar BCRs, at a higher rate than statistically expected, indicating a non-random distribution, probably due to chronic antigenic stimulation (7). Certain stereotypic BCR are associated with a poor prognosis (8). Fluorescence In Situ Hybridization (FISH), allows identification of the main CLL-associated cytogenetic abnormalities. About 80% of CLLs are associated with at least one of the four most frequent anomalies: deletion 13q (del 13q), deletion 11q (del 11q), deletion 17p (del 17p), and trisomy 12, encompassing miRNA 15a/16-1 (del 13q), *ATM* and *BIRC3* (del 11q), or *TP53* (del 17p). These abnormalities define different prognostic subgroups (9). The advent of Single Nucleotide Polymorphism (SNP) array allowed the discovery of smaller and less frequent Copy Number Variations (CNV) (10, 11). Next generation sequencing techniques made it possible to precisely define the CLL mutational landscape. This appears to be highly heterogeneous regarding pathway deregulation mechanisms, with a broad spectrum of mutations affecting: i) response to DNA damage and cell cycle control (*TP53, ATM, POT1, ATRX*), ii) RNA maturation and export (*SF3B1, XPO1, RPS15, DDX3X, ZNF292, MED12, NXF1*), iii) NOTCH pathway (*NOTCH1, FBXW7*), iv) BCR pathway (*EGR2, KLHL6, BCOR, IRF4, IKZF3, ITKB, CARD11*), v) chromatin remodeling (*CHD2, BAZ2A, SETD2, ASXL1, ZMYM3, HIST1H1E, ARID1A*), vi) NFKB pathway (*BIRC3, MYD88, TRAF3, NFKB1E*), vii) inflammatory response (*SAMHD1, RIPK1*), viii) early B-cell development (*IKZF3, PAX5*), ix) MAPK-ERK pathway (*MAPK, MAP2K1, ERK, BRAF, KRAS*), and x) MYC-associated signaling (*MGA, PTPN11*). The distribution and frequencies of these abnormalities are different among U-CLLs and M-CLLs (11–15).

Richter Syndrome (RS) is defined as the transformation of CLL or Small Lymphocytic Lymphoma (SLL) into a more aggressive histology (16). Two histopathological variants are described: Diffuse Large B-Cell Lymphoma (DLBCL, for 90%–95% of cases) and Hodgkin Lymphoma (HL, for 5-10% cases). Here we will focus on the DLBCL subtype. In more than 90% of cases, RS presents the immunohistochemical profile of non-Germinal Center-like DLBCL (17–22). RS incidence is very variable, ranging from 2% to 9% of unselected CLLs to more than 20% in the case of refractory and/or 17p deleted CLLs. Cumulative impact is estimated at 2.1% at 5 years and 4.8% at 10 years, representing a transformation risk of 0.5% per year. Median time between CLL diagnosis and RS transformation is 23 months (7, 19, 20, 22). Certain abnormality combinations systematically lead to the CLL transformation into RS, like the co-occurrence of an activating mutation of *NOTCH1*, trisomy 12, and an *IGHV*_4-39_ stereotypic BCR. Combination of an *SF3B1* mutation and a stereotypical BCR of the *IGHV*_3-21_ type leads to an increased risk of transformation into RS (23). Influence of CLL treatment on the risk of RS transformation is not established as half RS cases occur in the context of an untreated CLL. CLL treatment is associated with a RS risk of 1% per year (5% at 5 years, 15.2% at 10 years) (19). In a large retrospective study, a combination of purine analogs with alkylating agents increased the transformation risk to 1.5% per year. But the use of these different molecules in monotherapy (purine analog, alkylating agents or immunotherapy) does not increase RS risk (22). The impact of conventional chemotherapy on the risk of developing RS remains controversial because this risk is equivalent with Fludarabine alone, Chlorambucil alone or a combination of the two treatments (24). Similarly, after a median follow-up period of 3.5 years, the RS risk was equivalent between Chlorambucil alone, Fludarabine alone and the combination of Fludarabine + Cyclophosphamide (25). Recently, CLL management has been considerably modified with the availability of new BCR (26, 27) and B-cell Lymphoma 2 (BCL2) inhibitors (28). Unfortunately, these drugs do not preserve from RS, accounting for 30 to 50% of CLL progression cases. Transformation is now the principal obstacle for long-term control of the disease and remains a crucial unmet medical need (29, 30).

## Richter Syndrome Molecular Events

Exome sequencing shows that RS mutational landscape shares only a few common features with DLBCLs, except for *CARD11*, *MYD88*, *CDKN2A/B*, and *MYC* alterations (23, 31, 32). The genomic complexity of RS is intermediate between that of CLL and *de novo* DLBCL (32). Surprisingly, 64.7% of RS harbors an unmutated *IGHV* sequence, all *de novo* DLBCLs having a mutated *IGHV* profile. This is in line with the fact that U-CLL have a four-time higher RS transformation risk than M-CLL (33). RS exhibits an *IGHV* hypervariable CDR3 region identical to that of the initial CLL in 80-90% cases, proving a clonal relationship between the two stages (7). These clonally related RS have a median survival of 14.2 months. In contrast, the 10 to 20% clonally unrelated RS have a median survival comparable to *de novo* DLBCLs (62.5 months) and are considered by most authors as independent neoplasms (20, 21). Clonal relationship is therefore the most significant prognostic factor. Half RS harbor a stereotypic BCR (20), with an overrepresentation of *IGHV*_4-39_ in RS, suggesting a relationship with disease development.

*TP53* disruptions (partial or total deletions of the gene, loss of function mutations) are highly frequent at RS stage, with a prevalence of up to 34.4%–60% of cases in documented large cohorts (33). In most cases, *TP53* disruptions are acquired at RS transformation (20). In a large cohort of 131 RS patients, 45 (34.4%) had del (17p) or *TP53* mutation (34). The high proportion of these abnormalities at RS stage could reflect a selective advantage and the conferred chemoresistance. TP53 pathway is also disrupted through other abnormalities affecting related effectors such as *MDM2, MDM4, ATM, BCL2, CREBBP*, and *PRDM1* or *TP53* promoter hypermethylation (35).

*NOTCH1* mutations located in exon 34 are identified in up to 30%–40% of RS cases (36). Dominant positive variants devoid of the PEST degradation domain lead to a NOTCH1 protein with extended lifespan and a constitutive pathway activation, constantly triggering the transcription of many genes involved in cell proliferation and therefore uncontrolled cell growth. Trisomy 12 is present in 30% of RS and is frequently associated with *NOTCH1* mutations. Other RS recurrent abnormalities lead to NOTCH pathway deregulation, such as *NCOR1* deletions, *SPEN* mutations, *NLK* deletions, and *MYCN* amplifications.

*MYC* abnormalities, whether translocations or amplifications, affect 26.5%–35% of RS and are acquired at RS stage in 75% of cases. *MYC* and *TP53* abnormalities are described together in 50% of RS cases (20, 32, 34). An SNP study on a group of 13 RS, 8 of which were clonally related to the initial CLL, identified deletions of miR 17-92, a microRNA cluster regulating *MYC* expression. These anomalies are also acquired at RS stage (31). Last, other MYC pathway effectors are affected in RS, including deletions of negative regulator *MGA*, and mutations of *PIM1* and *PAX5*.

The study of CNVs by Comparative Genomic Hybridization array identified 9p21 deletions in 30% of RS cases. These are systematically acquired at transformation and encompass *CDKN2A/B* genes, encoding distinct negative regulators of cell cycle progression through inhibition of cyclin-dependent kinases 4 and 6 and cyclin D. Silencing through gene promoter hypermethylation is also described (35). These anomalies are bi-allelic in 30% of cases. This study confirmed the large proportion of linear evolutions of the CLL clone and the acquisition of an average of 22 new anomalies between the CLL and RS stage (36).

In the current molecular model, the evolution of the CLL clone into RS is associated with deregulation of cell proliferation, apoptosis and the cell cycle progression, mainly due to abnormalities of *TP53*, *NOTCH1*, *MYC*, and *CDKN2A/B* (37). Half RS cases are associated with the newly acquired *TP53* inactivation, *MYC* activation or *CDKN2A/B* deletion. In 30% of cases, the transformation is associated with trisomy 12 and a mutation of *NOTCH1*.These anomalies are mutually exclusive with *TP53* or *CDKN2A/B* disruptions. The remaining 20% RS are related to other genetic abnormalities (32, 36, 38).

**Figure 1:**
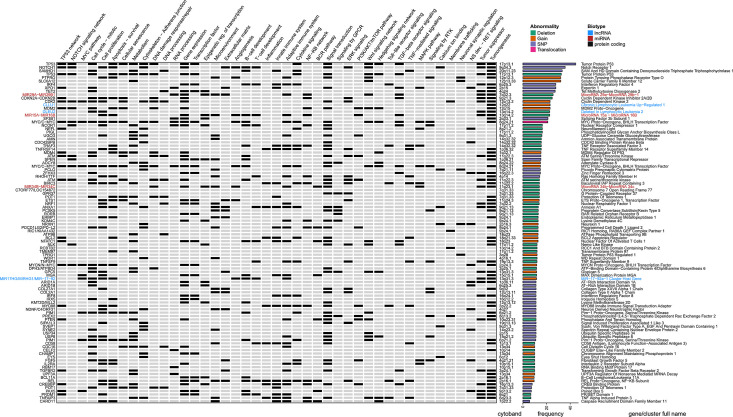
Genomic landscape of Richter syndrome. Genes recurrently mutated and related cellular functions altered in RS as a manually curated heat map of available data from 4 landmark papers, gathering a total of 207 RS patients. From left to right (for each gene): gene name, functional annotations and related pathways (according various databases and literature), cytogenetic band, nature of the reported abnormality (deletion, gain, translocation, or single nucleotide variant) and full gene name written according to a color-code indicating biotype (black: protein coding gene; red: miRNA and blue: lncRNA). Selection thresholds: 5% occurrence in cohorts of at least 50 samples for CNVs and 10 samples for SNPs. BCR, B-cell Receptor; CNV, Copy Number Variation; GPCR, G Protein-Coupled Receptor; lncRNA, Long Non-Coding RNA; MAPK, Mitogen-Activated Protein Kinase; miRNA, MicroRNA; NS, Nervous System; RS, Richter Syndrome; RTK, Receptor Tyrosine Kinase; SNP, Single Nucleotide Polymorphism; TGF, Transforming Growth Factor; TNF, Tumor Necrosis Factor.

To get a deeper understanding about RS genomic characteristics, we compiled the available data regarding DNA mutations from articles aiming at expanding knowledge about RS genomic features by exploring previously unknown genomic alterations on unselected cases. Richter cohorts documented with only previously known CLL-associated genomic alterations were not retrieved. This led us to select 4 landmark papers, gathering a total of 207 RS (20, 31, 32, 36) and retained: i) the CNVs described in at least 5% cases (with at least five positive cases and series of 50 samples minimal) and ii) SNPs described in at least 5% cases (with at least two positive cases and series of 10 samples minimal). This led to a list of 100 abnormalities affecting 95 different genes, which were annotated functionally with a compilation of gene ontologies (http://geneontology.org/) (39) and enriched pathways according to Reactome (https://reactome.org/) (40) and retrieved with BioMart (https://www.ensembl.org/biomart) (41). We also added the corresponding literature from OMIM (https://omim.org/) (42), and extensive lists of transcription factors obtained from a recent review on the topic (43), together with tumor suppressor candidates from the Tumor Suppressor Database (https://bioinfo.uth.edu/TSGene/) (44). All these were manually curated and used to complete a detailed heat map of RS genomic abnormalities (**Figure 1**).

According to this compilation, a wide range of cellular functions are deregulated in RS, including cell cycle regulation, cell proliferation, cell survival, senescence, DNA and RNA processing, epigenetic regulation of transcription, nuclear export, signal transduction, ion transport, cytoskeleton, cell adhesion, and migration, as well as numerous essential signaling pathways, implicating TP53, NOTCH, MYC, BCR, NF-KB, JAK-STAT, Toll-like receptors, MAPK, TNF, TGF, Insulin like growth factor, Wnt, Ras, ERK, and PI3K-Akt-mTOR. However, RS mutational landscape does not only affect cell cycle-related functions. Indeed, the alterations described may also disturb the microenvironment, B-cell development, and T-cell expansion/activation, promoting tumor progression by immune response reprogramming.

PTPRO (protein tyrosine phosphatase receptor type O) has been described as highly expressed in the microenvironment of breast cancer, associated with increased tumor growth, angiogenesis, and metastatic spreading (45). It also plays a role in T-cell-mediated anti-tumor immunity, with involvement in regulation of effector T-cells/regulatory T-cells ratio in the tumor microenvironment (46). Besides their known tumor suppressive properties in CLL, miR-29 family members are also regulators of the adaptive immune system, since they regulate helper T-cell development and interferon gamma (IFNγ) secretion by Type 1 helper T-cells (47). Another micro-RNA cluster, miR-15a/16 does not harbor functions restricted to tumor suppressive properties, since it also regulates T-cell expansion and differentiation (48), B cell proliferation (49), contributes to the balance between T-cell activation and T-cell anergy (50), regulates PD-1 (Programmed Death 1) expression and IFNγ excretion by tumor-infiltrating CD8+ T-cells (51), and indirectly governs regulatory T-cell development (52). TGF-β (Transforming Growth Factor β) receptor deregulation through TGF-β receptor 2 abnormalities (53), decreased expression of surface HLA class II molecules due to CD58 locus deletions (54) and impairment of Tumor-Associated Macrophages related to *XPO1* and *NRF1* abnormalities (55, 56) may also represent another player in immune evasion in RS.

XPO1-blocking drug Selinexor has been shown to slow down tumor growth in murine primary central nervous system lymphoma by shifting TAM polarization from PD-1 expressing M2-like macrophages toward PD-1 low-expressing M1 macrophages, providing evidence for the role of microenvironment remodeling in lymphoma (56). The central role of immune checkpoint hijacking through PD-1/PD-L1/PD-L2 pathway deregulation in RS is supported by abnormalities of PD-L1 (Programmed Death Ligand 1) expression-regulating miR-34 cluster (57) and high proportions of alterations of *PDCD1LG2*, encoding PD-L2 (Programmed Death Ligand 2).

## Activation and Limitation of the Adaptive Immune Response: Immune Checkpoint Deregulation Through PD-1/PD-L1/PD-L2 Pathway Hijacking in Oncology

**Figure 2:**
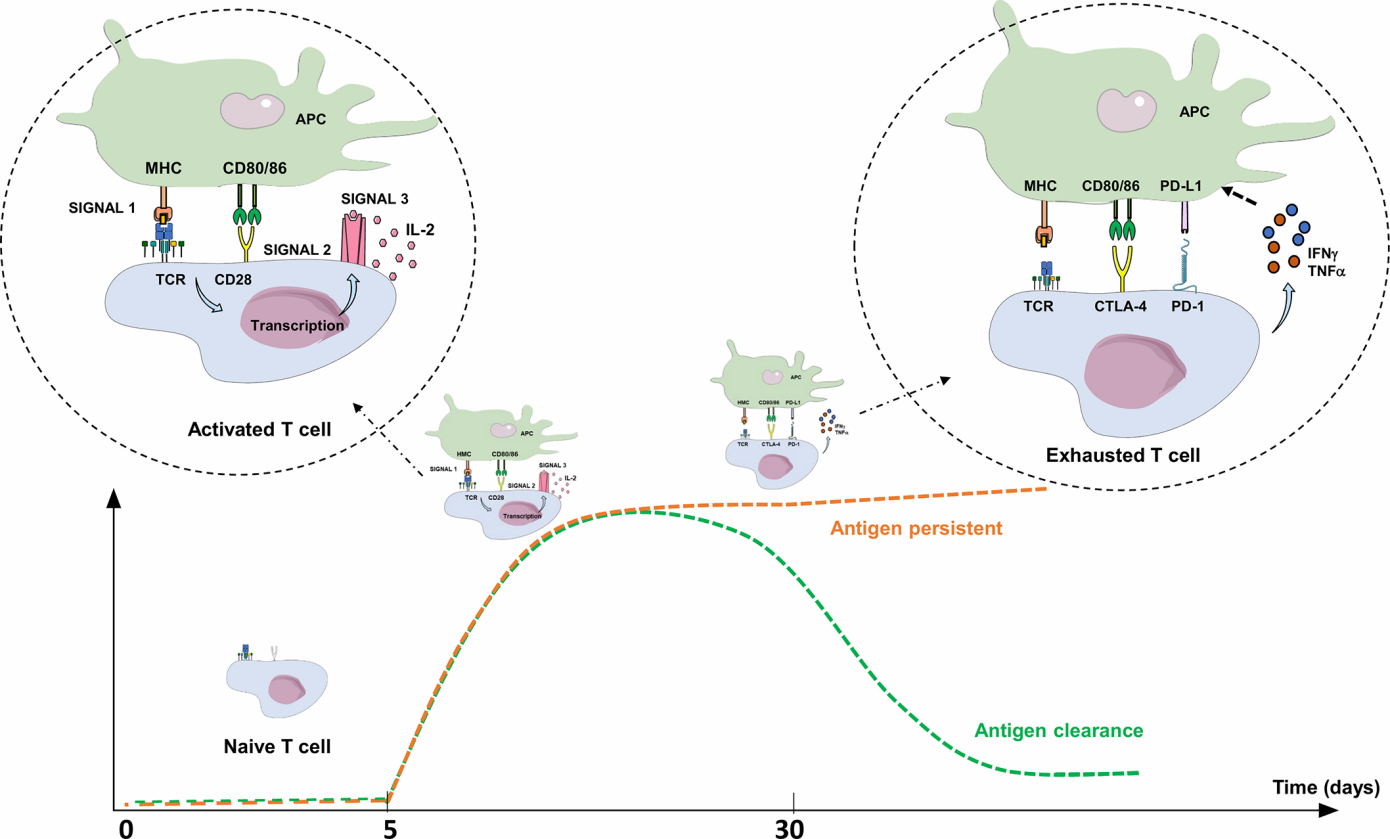
Activation and limitation of the adaptive immune response: physiological concepts. To activate a naive TL during adaptive immune response, APC must present an antigen through MHC to the TCR (signal 1), but also provide an essential co-stimulation signal (signal 2). These co-stimulation molecules, whose expression are induced during innate immune response, are mainly CD80/CD86 (expressed on APC), and CD28 (expressed on T lymphocyte). The activated TL can then proliferate, differentiate into an effector TL, leave the lymph node and move into peripheral tissues to the inflammatory site. Without this co-stimulation, the TL becomes anergic. To limit the immune response to a pathogenic antigen, both central and peripheral immune control checkpoints exist. Three to 5 days after activation, TLs physiologically express co-inhibitory receptors (immune checkpoint inhibitors; ICPIs) on their surface, such as CTLA-4 and PD-1. CTLA-4 is expressed by naive TLs residing in the lymph nodes, competes with CD28 and interacts with CD80/86: this regulates the amplitude of TL activation and limits the initial immune response. PD-1 is expressed on effector T lymphocytes and interacts with its ligands (PD-L1/PD-L2). PD-1 expression occurs within 24 h after T-cell activation and decreases with antigen clearance. Unlike CTLA-4, its role is to limit, through various signaling pathways, the immune response in peripheral tissues during the active phase of inflammation by decreasing TL activity (exhaustion). TL exhaustion is a progressive process consisting in an effector TL dysfunctional state upon repeated or prolonged stimulation by an antigen, happening in a context of chronic inflammation (chronic infection or cancer) with persistent TL stimulation. The expression of PD-L1 is induced by pro-inflammatory cytokines such as IFNγ or TNFα through a negative feedback loop, thus avoiding collateral damage on tissues. APC, Antigen Presenting Cell; CTLA-4, Cytotoxic T lymphocyte-associated Antigen 4; IFNγ: Interferon Gamma; IL-2, Interleukine 2; MHC, Major Histocompatibility Complex; PD-1, Programmed Death 1; PD-L1, Programmed Death Ligand 1; TCR, T-cell Receptor; TNFα, Tumor Necrosis Factor Alpha.

Three signals are required for T-cell/T lymphocyte (TL) activation: i) specific recognition by the T-cell receptor (TCR) of an antigen processed by professional antigen presenting cells (APCs), ii) co-stimulation signals, either through the binding of TL co-stimulatory receptor CD28 with its ligands, CD80 and CD86 expressed on APCs, or the binding of CD40 (receptor) to CD40L (ligand), and iii) TL cytokine production (IL-2 in particular) and expression of their specific receptors, leading to autocrine activation, clonal expansion, TL differentiation, and cytotoxic activity of antigen-specific TL. Co-stimulation is essential for TL activation, since antigen recognition by the TCR without co-stimulation signal leads to an anergy state and/or tolerance to this antigen (**Figure 2**) (58) Three to 5 days after activation, TLs physiologically express co-inhibitory receptors (immune checkpoint inhibitors; ICPIs) on their surface, such as PD-1, CTLA-4 (Cytotoxic T Lymphocyte-Associated protein 4), LAG-3 (Lymphocyte-Activation Gene 3), and TIM-3 (T-cell immunoglobulin and mucin containing protein-3), which bind to their respective ligand, leading to the regulation of the immune response. Once the antigen is eliminated, expression of these checkpoint inhibitors decreases to normal levels (**Figure 2**) (59, 60). The most explored immune checkpoint inhibitors to date are CTLA-4 and PD-1, both members of the B7 receptor family.

TL-specific surface marker CTLA-4 is a CD28 homologue expressed 48 hours after TL activation, with a greater affinity for CD80/86 (61). In lymph node, it acts as a central negative regulator on the surface of naive TLs (CD4+ FOXP3- CD8+), where it competes with CD28 and interacts with CD80/86, enabling the minute regulation of TL activation level, thus limiting early immune response. CTLA-4 is also expressed on CD4+ FOXP3+ regulatory T lymphocytes (Tregs) (62).

PD-1 is a transmembrane protein receptor that functions as a key negative regulator of cellular immunity, orchestrating the delicate balance between immune defense and the protection of healthy tissues from persistent inflammation and autoimmunity through various signaling pathways (59–61, 63–65). PD-1 is expressed on TL, but also on natural killer cells, pro-B cells, macrophages, monocytes, Dendritic Cells (DC), and Innate Lymphoid Cells (66), and has two ligands: Programmed Death Ligand 1 (PD-L1; CD274; B7-H1) and Programmed Death Ligand 2 (PD-L2; CD273; B7-DC). PD-L1 is more widely expressed than PD-L2 (67), notably on non-hematopoietic cells (including epithelial cells, vascular endothelial cells and stromal cells) and is induced by pro-inflammatory cytokines (including type I and type II interferons, TNFα and vascular endothelial growth factor). PD-L2 is mainly expressed on DC and macrophages and is induced by many of the same cytokines as PD-L1, plus IL-4 and granulocyte-macrophage colony-stimulating factor (59, 60, 64, 68). PD-1 expression occurs within 24 h after TL activation and decreases with antigen clearance. The binding of PD-L1 or PD-L2 results in TCR signaling downregulation. This inhibition induces a decrease in IL-2 and IFNγ production by activated TL, leading to a decrease in the downstream stimulation cascade, and a functional alteration of the CD4+ and CD8+ TLs, which in turn reduces their capacity to produce IL-2 and IFNγ, to acquire cytotoxic abilities and to proliferate (68, 69).

In a chronic inflammatory context, TL may enter a dysfunctional state called *lymphocyte exhaustion*, a progressive process consisting in an effector-TL function loss upon repeated activations, coinciding with increasing expression levels of ICPIs and particularly PD-1, considered as the leading inhibitory regulator of TL function. TL are ineffective at eradicating pathogens or tumors, so there is a real interest in reversing the depletion phenomenon (70).

**Figure 3:**
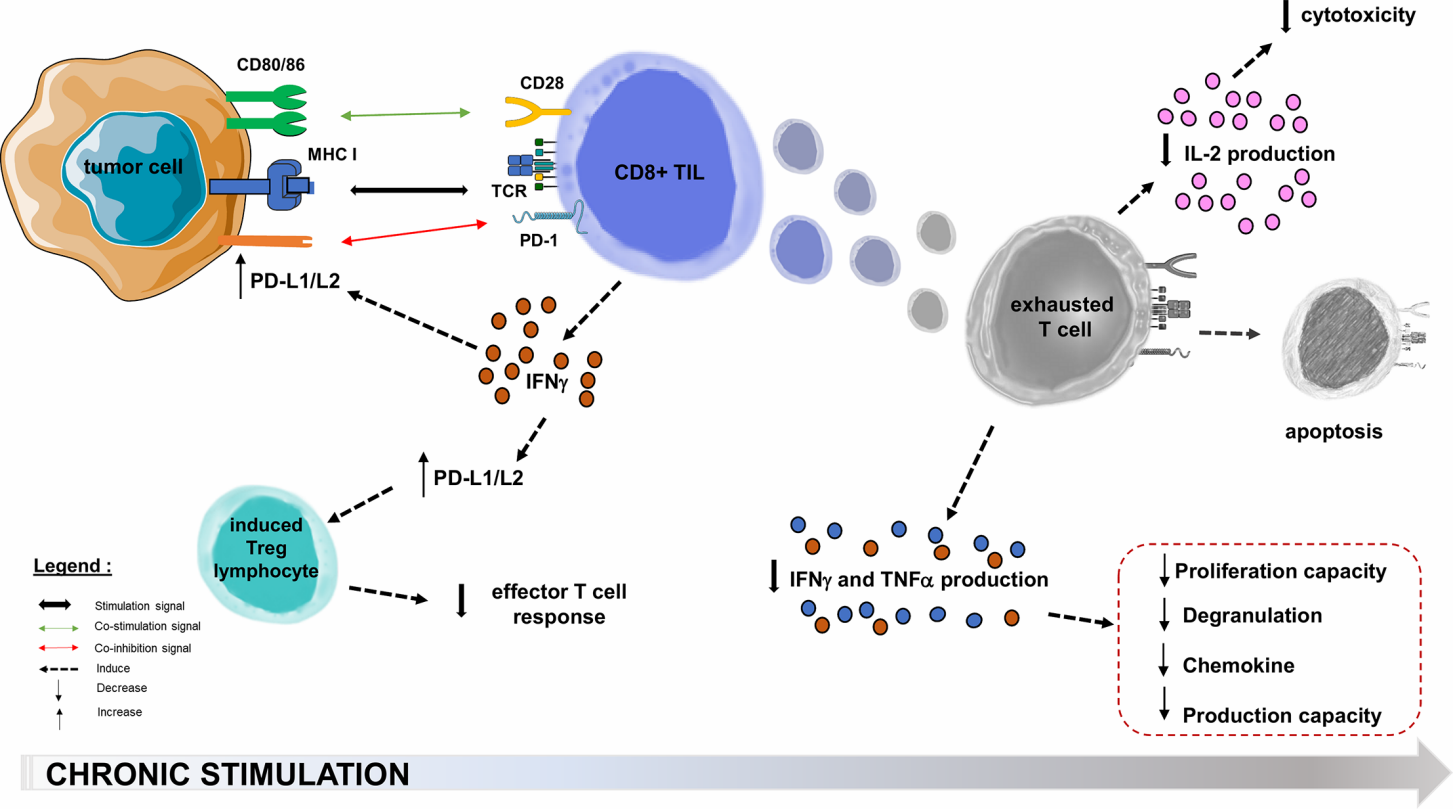
PD-1/PD-L1/PD-L2 pathway hijacking in oncology. Immune checkpoints are exploited by solid tumors to evade or suppress the immune system. The PD-1/PD-L1 co-inhibition axis, within the tumor microenvironment, will allow many PD-L1 tumor cells to escape the immune system through multiple mechanisms. One of these is the decrease of effector functions of cytotoxic TLs, directed against the tumor antigen by inducing their functional depletion (exhaustion), reducing their cytokines (IL-2) production ability, their high proliferation capacity, their cytotoxic activity and consequently the resistance to tumor cell lysis. The second step is the functional alterations in the production of TNFα, IFNγ, β−chemokines, and degranulation. In the most terminal stages of depletion, these cells may enter apoptosis, probably as a consequence of over-stimulation. PD-L1 role is also to maintain and induce tumor-associated regulatory T-cells (induced Tregs), by promoting their switching from naive CD4+ TLs. Infiltration of their microenvironment by activated TLs producing pro-inflammatory cytokines such as IFNγ enhances PD-L1 upregulation in tumor cells. This feedback loop is thought to be a mechanism of adaptive immune resistance by the tumor. APC, Antigen Presenting Cell; CTLA-4, Cytotoxic T lymphocyte-associated Antigen 4; IFNγ, Interferon Gamma; IL-2, Interleukine 2; MHC, Major Histocompatibility Complex; PD-1, Programmed Death 1; PD-L1, Programmed Death Ligand 1; TCR, T-cell Receptor; TIL, Tumor-infiltrating Lymphocyte; TNFα, Tumor Necrosis Factor Alpha; Treg, Regulatory T lymphocyte.

Oncogenic processes begin with an acute inflammatory response, with tumor infiltration by non-specific innate immune cells and increased production of pro-inflammatory cytokines, followed by an activating cascade specific of adaptive immune cells (71, 72). In the microenvironment of solid cancers, the co-stimulatory molecules regulating TL activation are not necessarily overexpressed. The inhibitory molecules that regulate TL functions (and notably the PD-1/PD-L1 axis) are generally overexpressed in tumor cells or in the microenvironment cells such as Tumor-infiltrating Lymphocytes (TIL) and Tregs in melanoma, breast, prostate, ovary, hepatocellular carcinoma and small cell lung carcinoma (65, 73). PD-1 overexpression in TILs affects the prognosis of several solid cancers (74), and the increased PD-1 expression among tumor-infiltrating CD4+ TLs reflects a usually high level of PD-1 expression on Tregs, which may represent a large proportion of intra-tumor CD4+ TLs (59). In a melanoma mouse model consisting in B16.SIY melanoma cells subcutaneously transplanted into immunocompetent 6-week old wild-type C57BL/6 mice, TL from the tumor microenvironment express very high levels of PD-L1 and indoleamine-2,3-dioxygenase, both induced by IFNγ production by CD8+ TLs (75). This adaptive tumor resistance mechanism uses large quantities of IFNγ from the tumor microenvironment (76, 77): such a negative feedback loop induces PD-L1 expression on the tumor cell surface, which in turn suppresses PD-1+ TLs activity (59, 63, 78). In many cancers, upregulation of PD-L1 appears to be correlated with poorer outcomes (**Figure 3**) (73). PD-L1 is also involved in the maintenance and induction of tumor-associated Tregs by inhibiting the Akt/mTOR signaling cascade, which promotes differentiation and switch from naive CD4+ TL to induced CD4+ CD25+ FOXP3+ Tregs (59, 62, 68, 79). The function of these Tregs is to attenuate the response of effector TLs and PD-1 (59, 78). By overexpressing PD-L1, tumor cells inhibit anti-tumor immune responses in the tumor microenvironment (59).

Blocking the PD-1/PD-L1 signaling pathway can therefore induce a targeted anti-tumor response (73). The presumed mechanism for blocking PD-1/PD-L1 in cancer is that it unleashes the anti-tumor TL response at the tumor site. To date, many monoclonal antibodies targeting PD-1 or its ligand has been approved by the US Food and Drug Administration (FDA) and are available with marketing authorizations in oncology (59, 63, 78, 80, 81).

## Immune Escape Through PD-1/PD-L1/PD-L2 Pathway in B-Cell Malignancies

**Figure 4:**
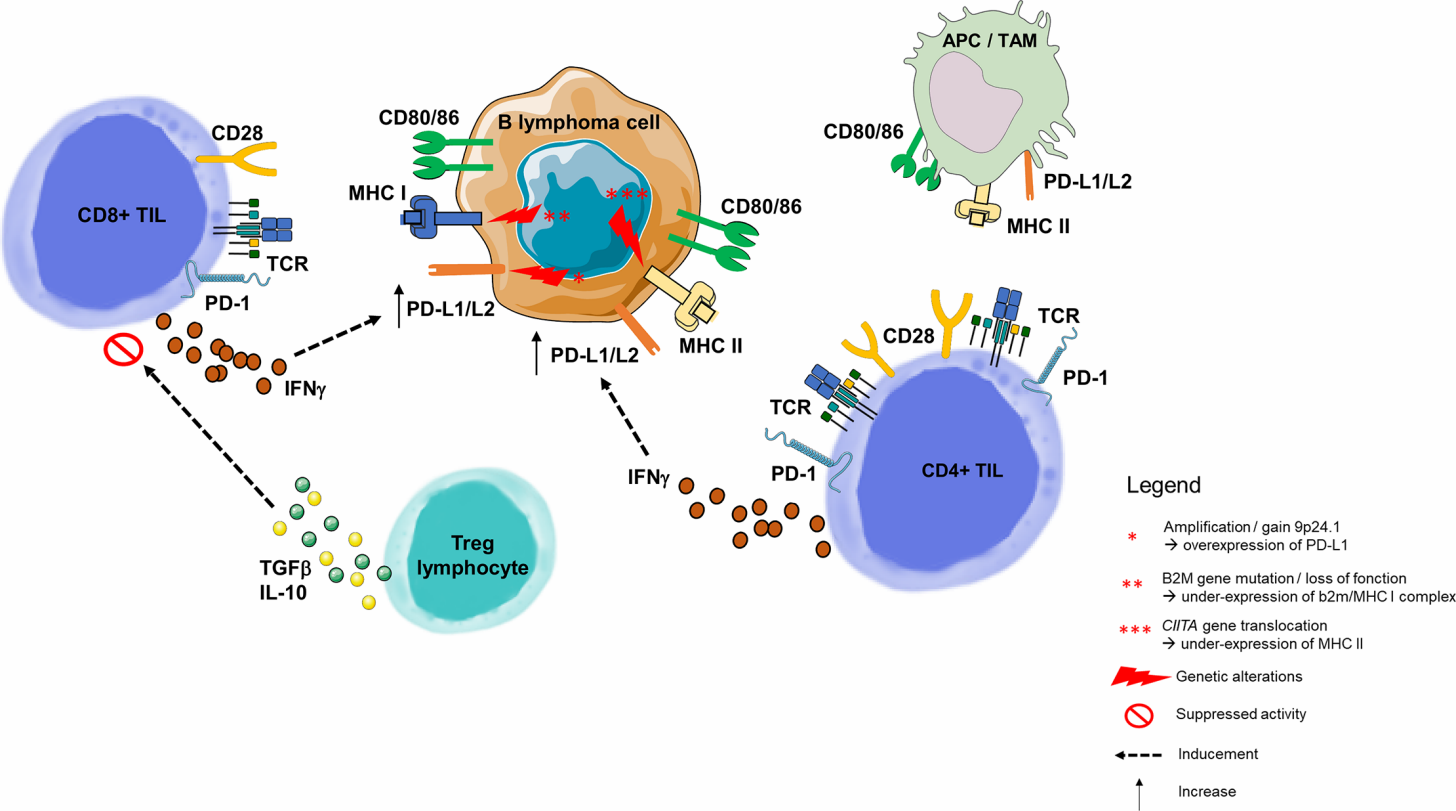
Immune escape through PD-1/PD-L1 pathway in B-cell malignancies. As opposed to solid cancers, the malignant B lymphoma cell is also an immune cell, and its tumor microenvironment contains highly variable numbers of immune cells. B-cell malignancies arise in lymphoid tissues, and more precisely germinal center of lymph nodes (82). Molecular dissection of the malignant B-cell allows to understand the progressive behavior of different B-cell lymphoma subtypes. Among genetic alterations contributing to immune escape is the 9p24.1 copy gain or amplification, described many times in inflammatory lymphomas such as HL or Primary Mediastinal B Lymphoma (84), and correlated with overexpression of PD-L1 and PD-L2 on the surface of malignant cells (85). Two other mechanisms responsible for the malignant B lymphocyte inability to present tumor antigen to the CD8+ or CD4+ TLs are, respectively, loss of function of the gene encoding the β2-microglobulin (MHC I complex dysfunction) (54, 86) and the dysfunction of *CIITA* (encoding MHC II) (87). PD-L1 is highly expressed on tumor-infiltrating macrophages and the surface of tumor cells and APCs in the tumor microenvironment (76). In a HL model, PD-L1+ macrophages were frequently in contact with PD-1 CD4+ TLs, suggesting that macrophages drive CD4+ TL dysfunction *via* PD-1/PD-L1 interactions, and/or by preventing direct access to Hodgkin Reed-Sternberg cells (88). Tumor cells upregulate PD-L1 to dampen cytotoxic TL attack. This upregulation is a consequence of pro-inflammatory cytokine production by tumor infiltrating immune cells: IFNγ is produced by CD4+ and CD8+ TLs and acts as a potent PD-L1 upregulator (76). In a CLL model, Beyer et al. observed a significantly increased expression of TGF-β and IL-10 in Tregs from patients. Both cytokines play an important role for the CD8+ TL inhibitory function of these cells (89). For example, in FL, malignant cells guide differentiation of CD4+ TLs, skewing the population within the tumor towards Tregs. APC, Antigen Presenting Cell; HL, Hodgkin Lymphoma; IFNγ, Interferon Gamma; IL-2, Interleukine 2; IL-10, Interleukine 10; MHC, Major Histocompatibility Complex; PD-1, Programmed Death 1; PD-L1, Programmed Death Ligand 1; PD-L2, Programmed Death Ligand 2; TAM, Tumor Associated Macrophage; TCR, T-cell Receptor; TGF-β, Transforming Growth Factor Beta; TIL, Tumor-infiltrating Lymphocyte; Treg, Regulatory T Lymphocyte.

The model used in oncology is not applicable here because the B lymphocyte tumor cell is also an APC (82, 83). This is because the tumoral microenvironment is usually the lymph node, where immune cells practically reside. In this environment, B lymphocyte interaction is different in each lymphoma subtype. Malignant B-cells attract non-malignant cells and modulate their plasticity, converting their environment into a supportive niche. It is therefore essential to understand how the dynamic interaction between these B-cells triggers the setting of a supportive niche, and consequently the mechanisms of B-cell immune escape (**Figure 4**) (90).

As in any adaptive immune response, lymphocyte activation requires the three signals described above (58). The B lymphoid tumor cell expresses class II MHC (Major Histocompatibility Complex) and the CD80/86 co-stimulation molecules which are functionally active and allow the tumor lymphocyte to act as an APC (83). An aberrant expression of PD-1 and its ligands PD-L1 and PD-L2 has been detected in many lymphoma subtypes, with a higher frequency for PD-L1. As was the case with solid cancers, PD-L1 reported levels vary highly between studies and within the same lymphoma subtype (58). Potential methodological issues may lead to this variability in PD-1 and PD-L1 expression measurements, and therefore, in the predictive value of these potential biomarkers. In particular, the detection method used (immunohistochemistry/IHC), flow cytometry or RNA sequencing), the specific antibody used for PD-1 or PD-L1 detection, the analyzed cell type, the sample type (bone marrow, blood, lymph node, peripheral organ), and the minimum threshold values to define PD-1 or PD-L1 expression are of consequence (58, 83). Thus, PD-L1 expression appears to be an imperfect predictor of PD-1/PD-L1 pathway inhibition efficacy, although response rates are significantly higher in patients with PD-L1-positive tumors.

Clinico-pathological studies (91–93) have investigated the expression of PD-1, PD-L1, and PD-L2 in a large number of tumor-cells or tumor-microenvironment-cells (403, 899, and 702 biopsies, respectively) in various B-cell neoplasms, whether Hodgkin or non-Hodgkin lymphoma: Burkitt Lymphoma, DLBCL, Follicular Lymphoma (FL), Mantle Cell Lymphoma, Marginal Zone Lymphoma, Primary Mediastinal Lymphoma, and CLL). In these studies, the expression of these tumor markers and their prognostic values vary in accordance with the lymphoma subtype.

## PD-1/PD-L1/PD-L2 Impairment in CLL

In CLL, malignant B-cells interact with neighboring cells in the lymph node, creating a microenvironment that promotes their proliferation and survival by inhibiting apoptosis and protecting them from immune system (94). One of the potential mechanisms responsible for immune escape from cytotoxic TLs in CLL is through PD-1 and both its ligands, PD-L1 and PD-L2. The neoplastic B-cells of the SLL/LLC lymph node weakly express PD-1 in most cases and series (95, 96), but more intensely and predominantly in prolymphocytes and paraimmunoblasts located in the proliferative centers of the lymph node. When expressing PD-1, circulating CLL cells also overexpress PD-L1, allowing tumor escape (94, 96, 97). Brusa et al. found a diffuse expression of PD-L1 in 9/20 samples of nodal B lymphocytes in CLL (96). This PD-L1 overexpression, by CLL B-cells exclusively, was comparable in the lymph node, the circulating blood and the bone marrow (94, 98). It was also significantly greater than that of B lymphocytes in healthy patients. This PD-1/PD-L1 increase is discordant in the literature, as some authors report no (91, 92) or feeble (92, 95) expression of PD-L1/PD-L2 on circulating CLL cells/lymph node SLL (**Table 1A** **).**

**Table 1:** PD-1 and PD-L1 expression changes on malignant B-cells and tumor microenvironment TL surfaces, in CLL (**Table 1A**) or RS (**Table 1B**) context.

A (CLL)									
Ref.	Number of patients	PD-1 expression				PD-L1 expression			
		Lymph node		Bone Marrow/Peripheral Blood		Lymph Node		Bone Marrow/Peripheral Blood	
		B-CLL	TIL	B-CLL	TIL	B-CLL	TIL	B-CLL	TIL
(95)	13	Yes	Yes	Yes (PB)	UD	No	UD	UD	UD
(96)	117	Yes	Yes	Yes (PB)	Yes (PB)	Yes	UD	Yes (PB)	UD
(94)	68	Yes	Yes	UD	UD	Yes	UD	Yes (PB)	UD
(99)	16	Yes	UD	UD	UD	UD	UD	UD	UD
(100)	39	Yes	Yes	UD	UD	No	UD	UD	UD
(91)	58	No	Yes	UD	UD	UD	UD	UD	UD
(98)	58	Yes	Yes	Yes	UD	Yes	UD	No	UD
(92)	37	UD	UD	UD	UD	No	UD	UD	UD
(97)	112	UD	UD	UD	UD	UD	UD	Only on MNC	UD
(101)	39	UD	UD	UD	Yes	UD	UD	UD	UD
(102)	16 (4 with tumor-invaded tissues)	Yes	Yes	UD	UD	Yes	Yes	UD	UD
(103)	18	UD	UD	UD	UD	No	UD	No	UD
**B (Richter Syndrome)**									
**Ref.**	**Number of patients**	**PD-1 expression**				**PD-L1 expression**			
		**Lymph node**		**Bone Marrow/Peripheral Blood**		**Lymph Node**		**Bone Marrow/Peripheral Blood**	
		**Richter cells**	**TIL**	**Richter cells**	**TIL**	**Richter cells**	**TIL**	**Richter cells**	**TIL**
(100)	15	Yes	Yes	UD	UD	Yes	No	UD	UD
(99)	17	Yes	UD	UD	UD	Yes	UD	UD	UD
(102)	9 (6 with tumor-invaded tissues)	Yes	Yes	UD	UD	Yes	No	UD	UD
(103)	15 (5 with tumor-invaded tissues)	UD	UD	UD	UD	Yes	No	UD	UD

PD-1, programmed death 1; PD-L1, programmed death ligand 1; B-CLL, CLL malignant B-cells; BM, bone marrow; LN, lymph node; MNC, mononuclear cells; PB, peripheral blood; RS-MO, RS microenvironment; TIL, tumor-infiltrating lymphocytes; UD, undetermined.

**Figure 5:**
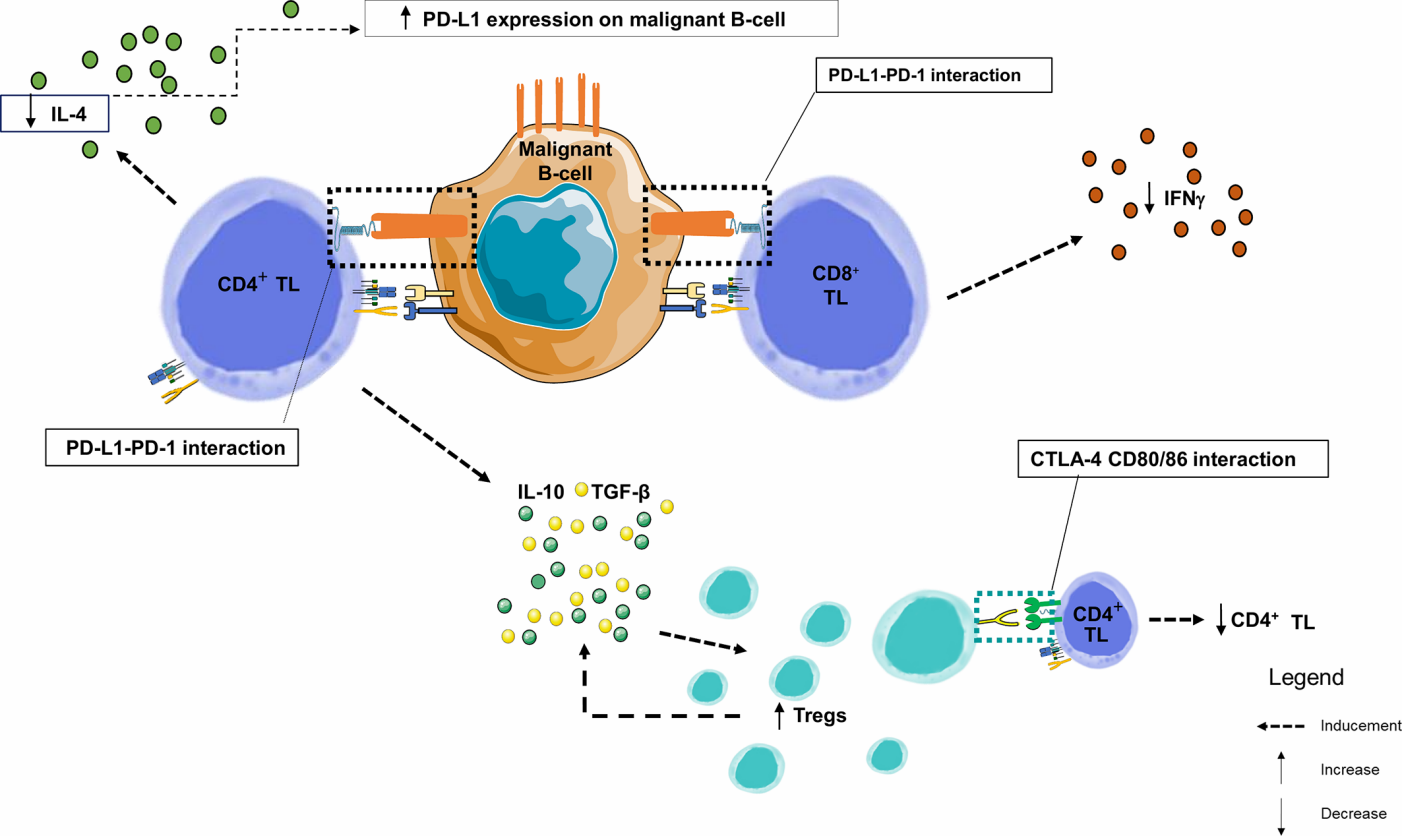
Immune synapse in a CLL node. This figure focuses on the germinal centers of lymph nodes. By combining data from several studies in lymph nodes and peripheral blood, we observe that PD-1 and PD-L1 are heterogeneously expressed markers of B lymphocytes from chronic lymphoid leukemia. Similarly, PD-1 is overexpressed in TLs infiltrating the CLL microenvironment. Within the malignant lymph node proliferation centers, PD-1+ CD4+ TLs are in close contact and interfere with PD-L1+ CLL leukemic B-cells. The number of CD8+ and CD4+ TLs are higher in CLL patients than in healthy donors, with an increase in effector TL number and a decrease in naive TLs. IFNγ production by TLs enhances PD-L1 expression. However, when PD-1 and PD-L1 interact, there is a significant decrease in IL-4 and IFNγ production by CD4+ and CD8+ TLs, respectively, creating a negative feedback loop, and a significant increase in the proportion of CD4+ CD25+ FOXP3 + CTLA-4+ Tregs in CLL patients. TGF-β and IL-10 are overexpressed by the CD4+ TL and Tregs and play an important role in their inhibitory function (106); IFNγ, Interferon Gamma; IL-4, Interleukine 4; IL-10, Interleukine 10; PD-1, Programmed Death 1; PD-L1, Programmed Death Ligand 1; TGF-β, Transforming Growth Factor Beta; TIL, Tumor-infiltrating Lymphocyte; TL, T Lymphocyte; Tregs, Regulatory T Lymphocytes.

In this neoplastic context, CD4+ and CD8+ TLs circulating or infiltrating the tumor exhibit exhaustion profile since they also express PD-1 (101), either slightly (1/4 cases) (91, 96) or significantly increased (104), and are in close contact with CD23+ CLL cells expressing PD-L1 within the lymph nodes. These CD4+ and CD8+ TLs exhibit the features of chronic activation, with an overexpression of CD69, HLA-DR and CD57 and an under-expression of CD28 and CD62L (104). Exhaustion markers CD244, CD160 and PD-1 (101). These exhausted TLs may be the result of chronic stimulation by low affinity auto-antigens. Due to exhaustion, CD8+ TLs lose their cytotoxicity and become unable to lyse target cells. However, in CLL, and unlike TLs exhausted after chronic stimulation by a high affinity viral antigen, CD8+ TLs keep their ability to produce IFNγ and Tumor necrosis factor alpha (TNFα), with normal IL-2 production potentially protecting CLL cells from apoptosis) (104). There is a specific cytokinic context in CLL proliferative centers where IFNγ production by TL promotes PD-L1 expression on leukemia cells. Conversely, PD-1/PD-L1 interaction triggers a negative feedback loop, with PD-1-mediated significant decrease in IL-4 and IFNγ production by CD4+ and CD8+ TLs, respectively (96). Investigations using human and murine CLL models showed alterations of the immunological synapse between tumor B-cells and CD4+ and CD8+ TLs, primarily due to the disorganization of the TL cytoskeleton through inhibition of TCR components by CLL cells (105). This leads to a decrease in TCR signaling and subsequent proliferation (decrease in production of IL-2 by CD4+ TL) and cytotoxic activity (decrease in cytokines) (**Figure 5**) (92, 101).

To study the CLL microenvironment, particularly the PD-1/PD-L1/PD-L2 axis, and to assess the functional impact of PD-1 expression on the effector function of TLs in CLL, different mouse models have been established (107). In these murine models (which compare an elderly mouse having spontaneously developed CLL to a young one with experimentally induced CLL, and to a healthy aged mouse), CD8+ PD-1+ infiltrating TLs retained their cytotoxicity capabilities but did not maintain a correct immunological synapse. IFNγ production was effectively altered but did not make these TLs exhausted. The ability of CD4+ TLs to switch from a naive phenotype to a memory TL phenotype after meeting the CLL tumor antigen was demonstrated in a similar mouse model, where CD4+ TLs that underwent phenotypic switching were able to protect tumor B-cells from apoptosis *in vitro*, and were associated with a more aggressive disease (106).

In addition to the increased expression of the PD-1/PD-L1 inhibitory receptors, an increase in CD4+ CD25+ FOXP3+ Tregs is observed in CLL, particularly in previously untreated advanced-stage CLL (89). Tregs also induce CTLA-4, another inhibitory receptor. Therefore, CTLA-4 signaling is likely to be another pathway mediating TL dysfunction in CLL.

## PD-1/PD-L1/PD-L2 Interplay Deregulation by Malignant B-Cells Promotes Immune Escape in RS

To date, few data are available on RS regarding the cellular interactions mediating the immunological synapse around the molecular surface marker PD-1 and its ligand PD-L1 (83, 104, 108). Here we will focus on PD-1 and PD-L1 deregulations in the context of RS, both on tumor B-cells and microenvironment cells, and the diagnostic and prognostic impact of the negative bi-directional interaction between these cells.

In a cohort of 80 patients including 39 CLLs, 15 RS, and 26 *de novo* DLBCLs, PD-1 expression in RS is significantly higher than in *de novo* DLBCLs. Only prolymphocytes and paraimmunoblasts from proliferation centers expressed PD-1 in CLL (100) and even more markedly in “accelerated” CLLs (109). A CLL-Richter clonal relationship, assessed by *IGHV* rearrangement comparison, has been recognized as an adverse prognostic factor in RS (19). In this cohort, He et al. (100). demonstrated that PD-1 expression by large B-cells from RS was highly correlated (90%) with clonally related RS. *IGHV* sequencing is the reference method for assessing CLL-RS clonal relationship (20, 100), but this test is expensive and dependent on the availability of the CLL component at RS diagnosis. It could advantageously be replaced by PD-1 estimation with IHC, which is more accessible for routine practice (101). In contrast, PD-L1 expression was only observed in 1/17 RS samples and 1/26 cases of *de novo* DLBCLs, in the surrounding immune environment consisting of histiocytes and DC.

Evaluation of PD-1 and PD-L1 expression on tumor B-cells from 10 biopsies (6 RS and 4 CLL) available beforehand showed a slight PD-L1 increase in patients with complete response (CR) or partial response (PR) after Pembrolizumab treatment and a tendency to PD-1 overexpression in these same patients *versus* non-responders (102). In tumor-infiltrating CD3+ CD8+ TLs, PD-1 and PD-L1 levels were similar in treatment responders *versus* non-responders. With confocal microscopy, PD-1 expression was observed mainly on tumor B-cells, while PD-L1 expression was observed on histiocytes/monocytes. Of note, FISH analysis did not find amplification or duplication of the 9p24 segment, which is the chromosomal location of PD-L1 and PD-L2. In another study, PD-L1 expression was high (> or equal to 5%) on 3 out of 5 evaluable RS biopsies (103). The study did not specify whether this expression was predominant on lymphoma B-cells or on cells of the microenvironment. PD-1 expression was not measured. These results were confirmed on a 58-patient cohort including 16 CLLs, 17 RS and 25 *de novo* DLBCLs (99), with i) a high correlation between CLL-RS clonal relationship and PD-1 expression (8/9 RS clonally related with matched CLL strongly expressing PD-1) and ii) a difference in PD-1 expression between RS (14/17 positive) and *de novo* DLBCL (2/25 weakly and locally positive). These results highlight the potential role of PD-1 in distinguishing RS from *de novo* DLBCLs or from a clonally unrelated RS. In line with what is described for CLL, PD-1 and PD-L1 expressions are variable within the tumor, either predominant on TIL or on malignant B-cells.

PD-1 positive B-cells in RS share the characteristics of the widely described regulatory B lymphocytes (Bregs) (110, 111), which interact with PD-L1-expressing immune-cells of the tumor stroma and subsequently with inhibitory cytokines (TGF-β and IL-10) from adjacent TL (**Table 1B** **).** Bregs are described as a B lymphocyte subtype representing less than 10% of total B-cells in a healthy patient but essential for the maintenance of immunotolerance (111). Consistent pre-clinical and clinical studies suggest several distinct Bregs phenotypes involved in autoimmune diseases and cancers (112). Immune suppression mediated by CD19+ CD1d+ CD5+ or CD19+ CD24+ CD3+ Breg subtypes is mediated by IL-10 production, which in turn inhibits Th1 cell activation, Th17 cell differentiation and promotes CD4+ TL conversion into suppressive Tregs. In metastatic hepatocellular carcinoma, a new Breg subtype has been identified within tumor PD-1+ B-cells, presenting a specific phenotype. In the course of hepatocellular carcinoma progression, PD-1 is strongly expressed by tumor-infiltrating B lymphocytes and interacts with PD-L1, expressed by tumor-associated macrophages, leading to IL-10 production by B lymphocytes, inhibition of TL cytotoxic activity and tumor expansion (110). This mechanism could synergistically work with the mechanisms of lymphocyte exhaustion mediated by the interaction of TLs expressing PD-1 with cancer cells expressing PD-L1. This observation is in accordance with similar results in thyroid cancers where tumor-infiltrating PD-1+ B-cells also express PD-L1. However, unlike PD-1+ Bregs, these PD-1+ B-cells do not increase IL-10 production and here, the immunosuppressive effect is mainly mediated by PD-L1 interaction with PD-1+ TLs (113). Several remaining hypothesis need to be explored: a) Bregs expressing IL-10 could also use this PD-1/PD-L1 pathway to neutralize TL activity and b) these Bregs could play an important role in B-cell lymphomas and more particularly in RS.

**Figure 6:**
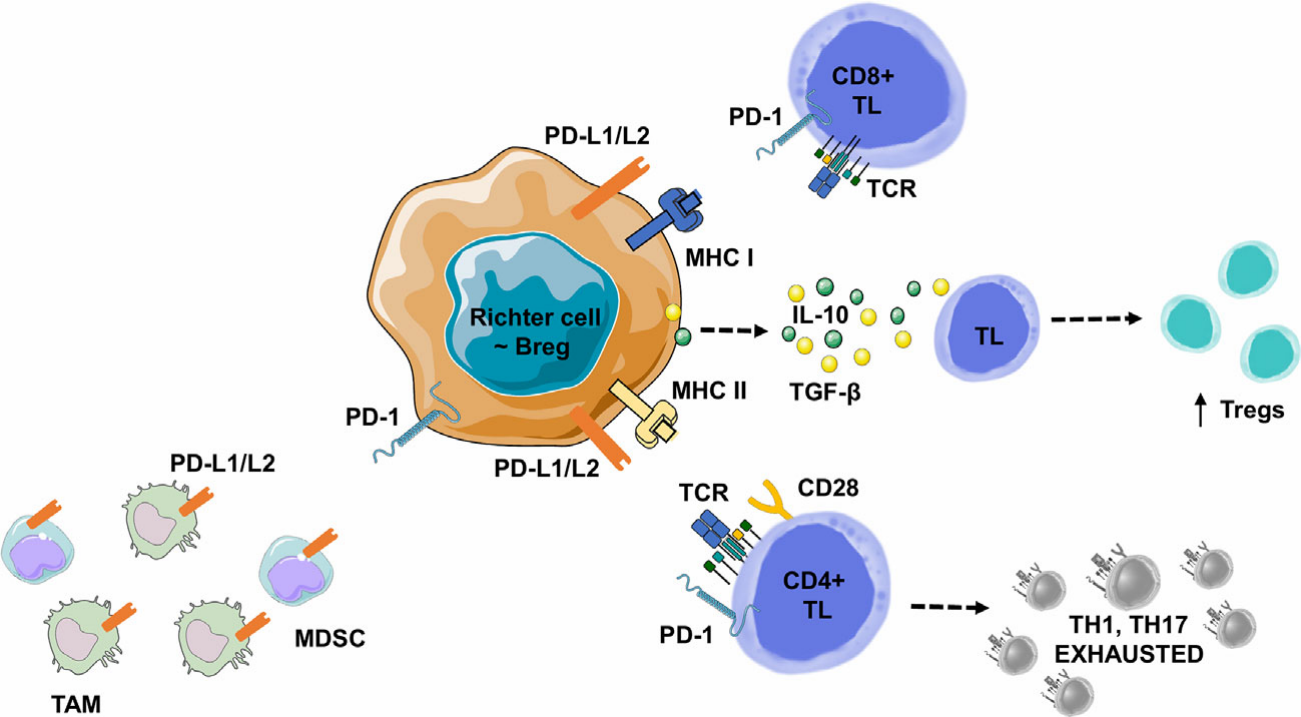
Lymphoma B cell-mediated immune synapse in Richter syndrome. This figure focuses on mechanism of tumoral escape in the germinal center of lymph nodes. PD-1 delivers inhibitory signals to TLs after binding with its ligands PD-L1 or PD-L2, on the surface of malignant B lymphocytes, in the tumor microenvironment. Behdad et al. (99). hypothesize that in RS, tumoral B lymphocytes share characteristics with Bregs: i) expression of co-inhibitory ligands such as PD-L1/PD-L2, allowing TL exhaustion, ii) the ability to induce FOXP3+ Treg expansion, and iii) the ability to recruit Myeloid-Derived Suppressor Cell (MDSC) or monocytes/macrophages (TAMs). Malignant B-cells can also directly express a variety of suppressive ligands and cytokines: i) TGF-β to promote Treg development and inhibit the differentiation of CD4+ TLs into Th1 or Th17 lymphocytes, and ii) IL-10 to promote CD4+ CD25+ FOXP3+ Treg development and Fas-L production (particularly for CD5+ B lymphocytes), leading to cell death *in vitro*. Bregs, Regulatory B Lymphocytes; IL-10, Interleukine 10; IFNγ, Interferon Gamma; MDSC, Myeloid-Derived Suppressor Cell; MHC, Major Histocompatibility Complex; PD-1, Programmed Death 1; PD-L1, Programmed Death Ligand 1; PD-L2, Programmed Death Ligand 2; RS-DLBCL, Richter Syndrome, Diffuse Large B-Cell Lymphoma subtype; TAM, Tumor Associated Macrophage; TCR, T-cell Receptor; TGF-β, Transforming Growth Factor Beta; TL, T Lymphocyte; Tregs, Regulatory T Lymphocytes.

In B-cell malignancies, tumor B-cells acquire Breg properties through different mechanisms, including the expression of co-inhibitory ligands, such as PD-L1/PD-L2, allowing TL exhaustion, but also the ability to induce FOXP3+ Treg expansion, to recruit myeloid-derived suppressor cells or monocytes/macrophages. Tumor B-cells can also directly express a variety of ligands and suppressive cytokines such as a) TGF-β, which promotes Treg development and inhibits CD4+ TL differentiation into Th1 or Th17, or b) IL-10 which promotes CD4+ CD25+ FOXP3+ Tregs development and CD5+ B-cell expression of Fas-L, leading to cell death *in vitro* (**Figure 6**) (112).

## Ensuing Therapeutic Options in RS

In B-cell neoplasms, the PD-1/PD-L1 axis has been widely explored. There is a correlation between PD-1 and PD-L1 expression levels and prognosis (58, 83, 91, 93, 108) as well as potential for therapeutic purposes (58, 74, 83, 114). The first indication in which PD-1 and PD-L1 inhibitors have been approved to date is relapsed or refractory classical HL after autologous stem cell transplantation (SCT) and treatment with Brentuximab-Vedotin. Nivolumab was approved by FDA in 2018 (115, 116). In the same indication, Pembrolizumab obtained approval in 2017 (117). Nivolumab is a human monoclonal IgG4 kappa anti-PD-1 antibody. Pembrolizumab is also a humanized monoclonal IgG4 kappa anti-PD-1 antibody, that is devoid of any cytotoxic activity because binding of pembrolizumab to PD-1 does not engage Fc receptors or activate complement. Nivolumab and Pembrolizumab block interactions between PD-1, which is a negative regulator of TL activation, and its ligands PD-L1 and PD-L2 (118). Numerous therapeutic trials concentrate on blocking the PD-1/PD-L1 pathway to modulate anti-cancer immunity are currently ongoing (76).

Improved knowledge on tumor microenvironment and particularly on the PD-1/PD-L1 axis in RS context, raises potential therapeutic options with targeted immunotherapies that blocks the interaction between PD-1 and its ligand to restore the activity of the tumor cell/TL immunological synapse. Effectiveness of immune checkpoint blockade therapies, including Pembrolizumab and Nivolumab, are demonstrated and approved for the treatment of many solid cancers (81), and relapsed or refractory HL (115, 116). Clinical trials are ongoing for other hematological malignancies (119).

In RS, three large cohorts of more than one hundred patients showed that median overall survival (OS) of RS patients is short, ranging from 5.9 to 12 months (34, 120, 121). In a large cohort of 103 RS patients, factors associated with RS development were del (17p), elevated thymidine kinase > 10 U/L, and presence of B-symptoms at the first-line treatment of CLL (119). The prognosis of RS patients who did not receive prior CLL therapy is better, with a median OS of 46.3 months versus 7.8 months (p < 0.001) (34). RS diagnosis is always an indication to start treatment. There is no randomized study comparing different therapeutic approaches in RS, but different treatment combinations have been individually tested. R-CHOP (Rituximab, Cyclophosphamide, Hydroxydaunorubicin, Oncovin, Prednisone) or R-CHOP-like regimens are widely used as a first-line option, with a response rate of around 67% (7% CR rate) but a progression-free survival (PFS) of only 10 months and a median survival of 15 months in eligible patients (38, 122). More intensive OFAR-type chemotherapy protocols (Oxiplatin, Fludarabine, Aracytine, Rituximab) only resulted in a CR rate of 6.5% and a median survival of 6–8 months (123, 124). These first-line protocols achieved a PR or a CR of short duration in 10%–15% cases and offered a median survival of 12 months (27% survival at 3 years). SCT can improve remission duration, usually short with chemotherapy regimens. Consolidation by an autologous or allogeneic SCT in a small subset of patients selected for their response to chemotherapy makes it possible to obtain a longer survival (75% at 3 years) (19, 125). A European retrospective study has compiled series of patients treated by autologous or allogeneic SCT: at 3 years, relapse-free survival is 27% after allogeneic SCT and 45% after autologous SCT (the non-relapse mortality at 3 years is 26% and 12%, respectively). However, most patients (85%–90%) are unfit or do not achieve an adequate response to be eligible for transplantation (126). Improving response rate to frontline therapy remains critical and new therapeutic approaches, such as Bruton Tyrosine Kinase (BTK) inhibitors yielded encouraging results (127). In this context, immune checkpoint inhibitors could therefore have a great therapeutic interest in RS.

A monocentric phase 2 clinical study tested the humanized anti-PD-1 antibody Pembrolizumab in a small cohort of 25 patients (16 relapsed CLLs and 9 RS). In RS, global response rate was 44%, PFS duration was 5.4 months, and OS was 10.7 months *versus* 3.5 months after classical immunochemotherapy courses. Patients previously treated with Ibrutinib (4/6) were still in clinical response after 11 months. About 20% of hematological adverse events above grade 3 were observed (102). However, none of the 16 CLL patients responded to Pembrolizumab. Notably, and despite a partial RS response, 3 CLLs progressed. Outside clinical trial, 10 patients with active RS and without new therapeutic options were treated either by Pembrolizumab (n=3) or by Nivolumab (n=7). This “real life” experience showed a time to treatment failure of 1.2 months, with 9/10 patients relapsing (128).

Nivolumab was used in combination with Ibrutinib in a phase II trial on 13 patients, including 5 relapsed or refractory (R/R) CLLs, 5 RS and 3 persistent CLLs after 9 months of Ibrutinib. This study showed PR and CR in 3 and 2 RS, respectively. However, this treatment had minor effects on CLL, with 1 CR and 3 PR in R/R CLLs and none in persistent CLLs (129). The Ibrutinib + Nivolumab association was tested in a second therapeutic trial conducted in 2 steps on 141 patients (103). The first phase (n=14) aimed at evaluating safety and toxicity parameters of the combined Ibrutinib + Nivolumab, administered to patients with high risk R/R CLL/SLL with del(17p) or del(11q), *de novo* DLBCL or FL. The main objective of the second phase (n=127) was to investigate the preliminary activity of this association in 4 patients subgroups: a) 36 high risk R/R CLL/SLL with del (17p) or del (11q); b) 40 FL; c) 45 *de novo* DLBCL and d) 20 RS. None of the 20 RS patients had been previously exposed to Ibrutinib as part of the underlying CLL. In the RS cohort, grade 3-5 adverse events were mainly hematological. 11/141 (8%) patients died, including four from the RS cohort, but none were attributable to Nivolumab-Ibrutinib. In RS, the overall response rate was 65% (13/20), including 10% (2/20) CR and 55% (11/20) PR. One patient had a stable disease and 5 patients (25%) progressed. PFS was 5 months and OS 10.3 months for a median follow-up of 8.7 months; 11 patients either progressed (n=3) or died (n=8). In summary, clinical responses were observed in all cohorts, but the overall response rate was the highest in the RS group. Of note, no CR was observed in the CLL/SLL cohort, but only PR (22/36, 61%). Although the treatment under evaluation was an anti-PD-1, Younes et al. (103) measured PD-L1 expression in five patients (out of 15 available RS samples). PR associated with prolonged OS was observed in the 3/5 patients who had a high (> or equal to 5%) PD-L1 expression, in line with previous results (**Table 2**) (102).

**Table 2:** Published clinical studies in RS.

Ref	Drugs	Study features	Number of patients	Schedule	CR (%)	ORR (%)	OS (months)	PFS (months)	Grade 3/4 adverse events
(102)	Pembrolizumab	Phase II unicentric clinical trial	25, including: - Relapsed CLL: 16 - RS: 9	200 mg/ 3 weeks	11	44	10.7	5.4	Thrombocytopenia (20%) Anemia (20%) Neutropenia (20%) dyspnea (8%) Hypoxia (8%)
(128)	Pembrolizumab or Nivolumab	Single center experience	10 RS: Nivolumab: 7 Pembrolizumab: 3	Doses according to their US label	10	UD	4.2	1.2	UD
(129)	Nivolumab and Ibrutinib	Phase II clinical trial	13, including: - Relapsed/ refractory CLL: 5 - CLL treated with ibrutinib for ≥ 9 months with persistent disease: 3 - RS: 5	Nivolumab 3mg/kg/15 weeks (cycle 1) Nivolumab 3mg/kg/15 weeks + Ibrutinib 420mg/day (cycle 2)	60	UD	UD	UD	/
(103)	Nivolumab and Ibrutinib	Phase 1/2a open-label multicentric clinical trial	141, including: - CLL/SLL: 36 - de novo DLBCL: 45 - FL: 40 - RS: 20	Nivolumab 3mg/kg / 15weeks + Ibrutinib 420 or 560 mg/day.	10	65	10.3	5	Neutropenia (25%), Anemia (35%), Thrombocytopenia (10%) Pneumoniae (10%) Rash (10%) Dyspnea (10%) hypotension (15%)

CLL, chronic lymphocytic leukemia; CR, complete response; DLBCL, diffuse large B-cell lymphoma; FL, follicular lymphoma; ORR, overall response rate; OS, overall survival; PFS, progression-free survival; SLL, small lymphocytic lymphoma; RS, Richter syndrome; UD, undetermined.

PD-L2 is expressed in a large panel of cancers and is upregulated in various B-cell lymphoma subtypes (130, 131). This may explain PD-1 inhibitors efficacy in the context of PD-L1 negative tumors. In addition, PD-L2 expression in tumor tissues is significantly associated with PFS under Pembrolizumab treatment, regardless of PD-L1 expression (132). On the other hand PD-L2 expression is involved in resistance to anti-PD-L1 monotherapy. In this context, antitumor immunity can be restored either by replacing the anti-PD-L1 therapy by an anti-PD-1 therapy or by combining an anti-PD-L1 with an anti-PD-L2 treatment (133).

Numerous clinical trials are ongoing in RS, combining an anti-PD-1 antibody to other drugs (Jain N et al., NCT02846623 and NCT02420912; Eichhorst B et al., NCT04271956; Danilov A et al., NCT03884998; Ding W et al., NCT02332980; Woyach JA et al., NCT03892044; Acerta Clinical Trials, NCT02362035) or an anti-PD-L1 antibody (Tedeschi A et al., NCT04082897; Herrera AF et al., NCT03321643; Mato AR et al., NCT02535286). Most of these phase II studies are currently evaluating the toxicity and safety of these combinations, with efficacy endpoints as secondary objectives (**Table 3** **)**.

**Table 3:** Ongoing clinical trials in RS.

Reference	Drugs	Study features	Patients enrollment	Protocol	Primary outcome	Secondary outcome
NCT02846623	Atezolizumab + Obinutuzumab + Venetoclax	Phase II open label clinical trial	65 R/R CLL/SLL and RS	A + O + V for 14 cycles of 28 days vs A+ O + V for 25 cycles of 28 days.	Minimal residual disease negative rate	AEs, best ORR, CRR, duration of response, PFS, OS
NCT04271956	Zanubrutinib + Tislelizumab	Prospective phase II open label multicenter clinical trial	45 RS	T + Z for induction and consolidation (6 cycles each), then maintenance until DP or allo-SCT	ORR after induction according to the Lugano Classification	ORR after induction therapy (IWCLL criteria) ORR after consolidation therapy, PFS, OS, TTNT, duration of response, AEs
NCT03884998	Copanlisib+ Nivolumab	Phase I open label clinical trial	15 RS or Transformed Indolent NHL	C + N for 12 cycles of 28 days in the absence of DP or UT.	Incidence of dose-limiting toxicities & AEs	ORR, duration of treatment, PFS, OS
NCT02332980	Pembrolizumab + Idelalisib or Pembrolizumab + Ibrutinib or Pembrolizumab alone	Phase II open label clinical trial	68 R/R CLL or other low-grade B NHL	P for 12 cycles, or P + I or P + Id for 12-24 cycles in the absence of DP or UT.	Confirmed response	CRR, AEs incidence, ORR, PFS, survival
NCT03892044	Duvelisib and Nivolumab	Phase I open label clinical trial	44 RS or transformed FL	N. + D for 28 days cycles until DP or UT.	Maximal Tolerated Dose	ORR, PFS, OS
NCT04082897	Atezolizumab and Obinituzumab and Venetoclax	Phase II open-labeled, uncontrolled, multicenter clinical trial	28 RS	A + O + V from cycle 1 to 8; A + V from cycle 9 to 18; V only from cycle 19 to 35	ORR	AEs (CTCAE v4), CRR, duration of response, PFS, OS
NCT02420912	Nivolumab and Ibrutinib	Phase II open-labelled non-randomized clinical trial	72 R/R or high-risk untreated CLL, SLL, or RS	For RS: N+ I for 1 or 2-24 cycles if no DP or UT.	CR or CR with incomplete BM recovery	AEs (CTCAE v4), OS, PFS
NCT03321643	Atezolizumab and Rituximab and Oxaliplatin and Gemcitabine	Phase I open-label sign group assignment clinical trial	30 transformed DLBCL (including RS)	Induction: [R + Ox + Gem] + A starting cycle 2. Maintenance: R + A	AEs (CTCAE v5), Maximal Tolerated Dose	CRR, best ORR, biomarker analysis
NCT02362035	Acalabrutinib and Pembrolizumab	Phase Ib/II open label clinical trial	161 B-cell malignancies	UD	AEs	UD
NCT02535286	Ublituximab and Umbralisib and Cosibelimab	Phase I open label clinical trial	20 R/R CLL or RS	Ub followed by maintenance infusions of Um. + Cos	AEs	ORR
NCT03121534	Blinatumomab	Phase II open label clinical trial	10 RS	Induction 8 weeks. If objective response: consolidation 4 weeks	ORR	Toxicity
NCT03931642	R-CHOP and Blinatumomab	Phase II open label clinical trial	35 RS (DLBCL)	2 R-CHOP cycles then Bl if CR and no measurable lesion	CRR	AEs (CTCAE v4), OR, CR
NCT02924402	XmAb13676	Phase I open label clinical trial	66 non B-cell NHL, CLL/SLL/RS.	XmAb13676 administered weekly up to 8 weeks	AEs (CTCAE v4), max tolerated or recommended dose	/

A, atezolizumab (anti-PD-L1); Ac, acalabrutinib (BTK inhibitor); AEs, adverse events; CTCAE, Common Terminology Criteria for Adverse Events; allo-SCT, allogeneic stem cell transplantation; BM, bone marrow; Bl, blinatumomab (anti-CD19 and anti-CD3 bispecific antibody); BTK; Bruton tyrosine kinase; CLL, chronic lymphocytic leukemia; Cop, copanlisib (PI3Kα,δ inhibitor); Cos, cosibelimab (anti-PD-L1); CR, complete response; CRR, complete response rate; D, duvelisib (inhibitor of PI3Kδ and PI3Kγ); DLBCL, diffuse large B cell lymphoma; FL, follicular lymphoma; DP, disease progression; Gem, gemcitabine; I, ibrutinib (BTK inhibitor); Id, idelalisib (PI3Kδ Inhibitor); IWCLL, International Workshop on Chronic Lymphocytic Leukemia; N, nivolumab (anti-PD-1); NHL, non-Hodgkin lymphoma; O, obinutuzumab (anti-CD20); ORR, overall response rate; OS, overall survival; Ox, oxaliplatin; P, pembrolizumab (anti-PD-1); PFS, progression-free survival; R, rituximab (anti-CD20); R-CHOP, rituximab, cyclophosphamide, hydroxydaunorubicin, oncovin, prednisone; R/R, relapsed or refractory; RS, Richter syndrome; SLL, small lymphocytic lymphoma; T, tislelizumab (anti-PD-1); TTNT, time to next treatment; Ub, ublituximab (anti-CD20); UD, undetermined; Um, umbralisib (anti-PI3Kδ and CK1e); UT, unacceptable toxicity; V, venetoclax (anti-BCL-2); X, XmAb13676 (anti-CD20 and anti-CD3 bispecific antibody); Z, zanubrutinib (BTK inhibitor).

**Figure 7:**
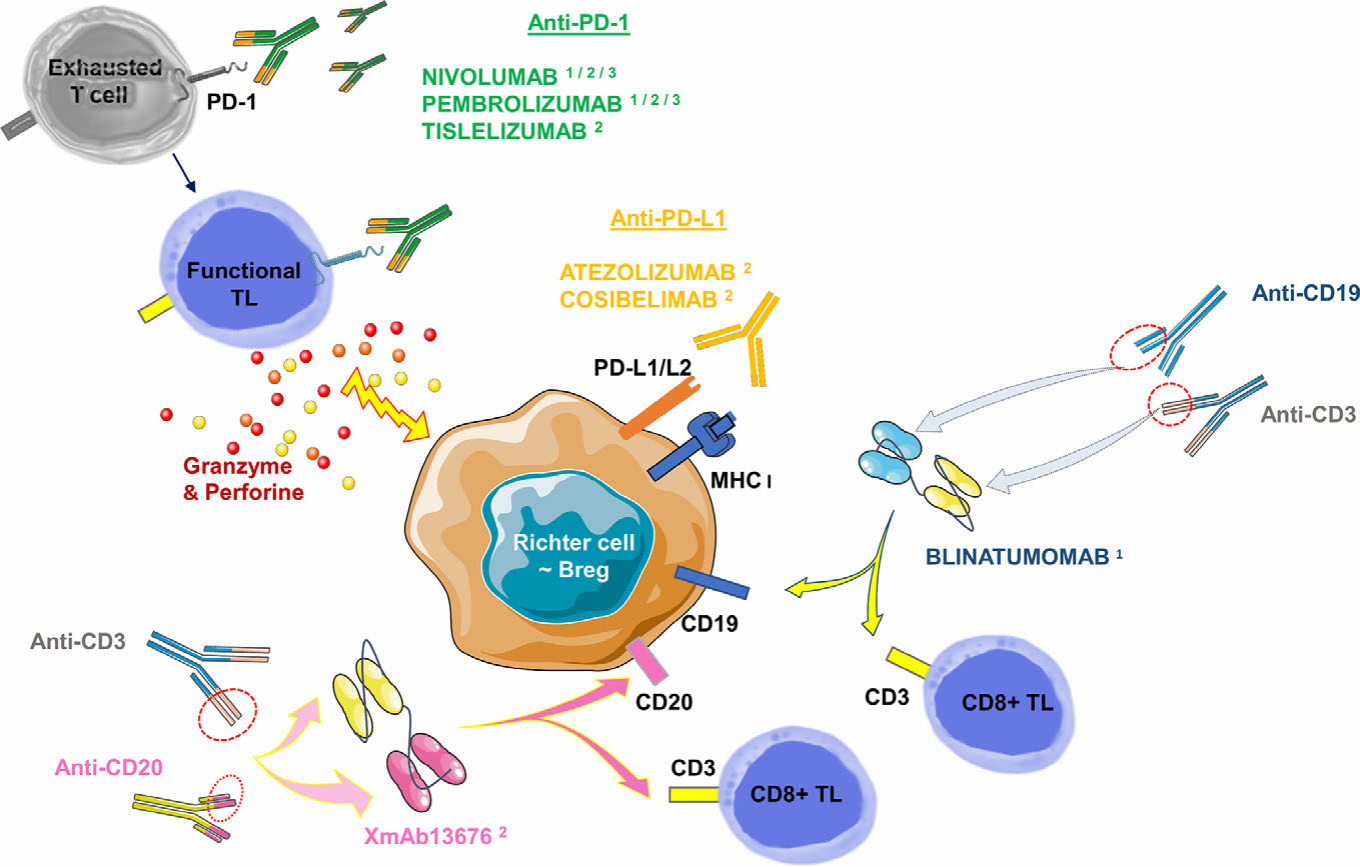
Monoclonal antibodies targeting the PD-1/PD-L1 axis (checkpoint inhibitors) and bispecific antibodies commercially available or in published/ongoing clinical trials in RS. Monoclonal antibodies targeting the PD-1/PD-L1 axis can be divided into two groups: i) against PD-1 receptor and ii) against its ligands PD-L1 or PD-L2. Anti-PD-1 antibodies are mainly represented by Nivolumab: a full human IgG4, and Pembrolizumab: a humanized IgG4. Tislelizumab (BGB-A317) is a new humanized IgG4 anti-PD-1 antibody. Anti-PD-L1 antibodies are mainly represented by Atezolizumab, a humanized antibody. Cosibelimab (TG-1501) is a novel, fully humanized anti-PD-L1 antibody. Bispecific antibodies are designed to direct cytotoxic TLs expressing CD3 towards B-cells expressing CD19 (Blinatumomab) or CD20 (XmAb13676). Breg, Regulatory B Lymphocyte; MHC, Major Histocompatibility Complex; PD-1, Programmed Death 1; PD-L1, Programmed Death Ligand 1; PD-L2, Programmed Death Ligand 2; TL, T Lymphocyte. ^1^: commercially approved in other hemopathies (relapsed Hodgkin Lymphoma for Nivolumab and Pembrolizumab, relapsed/refractory Acute B Lymphoid Leukemia for Blinatumomab). ^2^: clinical trials published in RS. ^3^: clinical trials underway in RS.

Other immunotherapies have already been successfully tested in hematological diseases such as bispecific CD19-CD3 antibodies (Blinatumomab). Bispecific antibodies transiently induce a synapse between target cancer B-cells and cytotoxic TLs, resulting in TL activation and lysis of the malignant B-cell. This molecule provided a CR at 35 days of treatment in a case report: it involved a 63-year-old man whose RS was refractory to two courses of R-ICE (Rituximab- Ifosfamide-Cisplatine-Etoposide) followed by a course of R-DHAP (Rituximab-Cytarabine-Cisplatine-Dexamethasone) + Ibrutinib. In the aftermath, he was able to benefit from an allogeneic SCT and remained in CR for 130 days of treatment. Of note is the occurrence of grade 3 encephalopathy on day 16, which resolved after a dose reduction (134). A phase II clinical trial evaluating the overall response rate and potential toxicity of Blinatumomab in 10 RS patients is currently underway and results will be available in 2021 (Thompson PA et al., NCT03121534). In the BLINART clinical trial, also currently underway (NCT03931642), Blinatumomab is administered after 2 courses of debulking R-CHOP in 35 RS cases. The hypothesis proposed is the improvement of the CR rate at 8 weeks of treatment. New bispecific antibodies are currently tested. An anti-CD20 combined with an anti-CD3 antibody (XmAb13676) is currently in a phase I study in two groups: patients with non-CLL B-cell malignancies and patients with CLL/SLL/RS (NCT02924402) (**Table 3**, **Figure 7**). Finally, Ipilimumab, an anti-CTLA-4 antibody, has been used in phase I studies in a few relapsed HL cases (135), and in R/R Non-Hodgkin Lymphoma cases (136). There is no ongoing clinical trial or published clinical trial about RS to date.

## Conclusion

A deeper characterization of RS is ongoing with the advent of novel sequencing and staining technologies, which can lead to a better understanding of this difficult-to-treat entity. Recent data enlighten the role of TL infiltration and immune system in RS specimens. TL exhaustion is driven in part by immune checkpoint deregulation, including high expression levels of checkpoint inhibitory molecules on TLs, such as PD-1, and expression of ligands for these molecules on CLL cells. Recent data showed that PD-1 expression by neoplastic B-cell was weak in both CLL and *de novo* DLBCL and strong in RS. Interestingly, this observation was linked to clinical responses to the PD-1 blocking antibody Pembrolizumab in RS, whereas no clear activity was observed in CLL. Furthermore, high tumor cell mutational burden is emerging as a predictor of improved therapeutic response to these agents. This could increase the neoantigens load at RS, leading to an immune response.

Exploring the expression of the PD-1/PD-L1/PD-L2 axis components in the immune ecosystem of RS at diagnosis is of importance, both for evaluating these potential biomarkers and to initiate a therapy specifically targeting the checkpoint inhibitors. These immunotherapies are already approved in relapsed HL and are currently under evaluation in multiple therapeutic trials focusing on B-cell malignancies. Positive results in CLL, DLBCL, FL, and HL make them prone to be included in future therapeutic strategies. Regarding RS, the expression of PD-1 and PD-L1 is variable according to the series, and the data are discordant concerning the location of these membrane proteins. Regarding therapeutic trials, they seem rather encouraging, with better overall response rates than in CLL. PD-1/PD-L1 level measurements using IHC should be systematic at RS diagnosis to tailor the use of these immunotherapies, already widely used in oncology in the context of new clinical trials precisely developed for this indication.

## Author Contributions

JB and PF supervised the manuscript. JB, HA, and RM selected and reviewed the papers. JB, HA, SH, and ABN made the figures and tables. JB, HA, SH, PF, and ABN wrote the manuscript. All authors contributed to the article and approved the submitted version.

## Conflict of Interest

The authors declare that the research was conducted in the absence of any commercial or financial relationships that could be construed as a potential conflict of interest.

## Abbreviations

APC, Antigen Presenting Cell; BCL2, B-Cell Lymphoma 2; BCR, B-Cell Receptor; Bregs, Regulatory B lymphocytes; BTK, Bruton Tyrosine Kinase; CLL, Chronic Lymphocytic Leukemia; CTLA-4, Cytotoxic T Lymphocyte-Associated protein 4; CNV, Copy Number Variation; CR, Complete Response; DC, Dendritic Cells; DLBCL, Diffuse Large B-Cell Lymphoma; FDA, Food and Drug Administration; FL, Follicular Lymphoma; HL, Hodgkin Lymphoma; ICPIs, Immune Checkpoint Inhibitors; IHC, Immunohistochemistry; IFNγ, Interferon Gamma; IGHV, Immunoglobulin Heavy chain Variable domain; IL-2, Interleukine 2; IL-4, Interleukine 4; IL-10, Interleukine 10; M-CLL, Mutated-CLL; MHC, Major Histocompatibility Complex; OFAR, Oxiplatin, Fludarabine, Aracytine, Rituximab; OS, Overall Survival; PD-1, Programmed Death 1; PD-L1, Programmed Death Ligand 1; PD-L2, Programmed Death Ligand 2; PFS, Progression-free Survival; PR, Partial Response; R-CHOP, Rituximab, Cyclophosphamide, Hydroxydaunorubicin, Oncovin, Prednisone; R/R, Relapsed or Refractory; RS, Richter Syndrome; SCT, Stem Cell Transplantation; SLL, Small Lymphocytic Lymphoma; TCR, T-Cell Receptor; TGF-β, Transforming Growth Factor Beta; TL, T Lymphocyte/T-Cell; TIL, Tumor-infiltrating Lymphocytes; TNFα, Tumor Necrosis Factor Alpha; Tregs, Regulatory T Lymphocytes; U-CLL, Unmutated CLL.
